# Unifying Field and Temperature Acceleration in Semiconductor Device Reliability Through Entropic Kinetics

**DOI:** 10.3390/mi17101116

**Published:** 2026-09-24

**Authors:** Liron Cohen, Lidor Bazon, Joseph B. Bernstein

**Affiliations:** The Department of Electrical and Electronics Engineering, Ariel University, Ariel 40700, Israel; lidorb@ariel.ac.il (L.B.); josephbe@ariel.ac.il (J.B.B.)

**Keywords:** time-dependent dielectric breakdown (TDDB), thermodynamic entropy, apparent activation energy, dielectric reliability, power-law kinetics, Gibbs free energy, field acceleration

## Abstract

Time-dependent dielectric breakdown (TDDB) is an important failure mechanism that affects semiconductor device reliability. The degradation lifetime depends strongly on both electric field and temperature, yet these dependencies are commonly treated using separate empirical acceleration factors. This work develops a thermodynamic description in which electrical stress changes the accessible microscopic states of the dielectric and therefore contributes an entropic term to the Gibbs free energy. The resulting formulation separates the field-independent activation barrier, $E_{a}$, from a field-dependent entropic contribution characterized by α. This relation predicts a linear decrease in apparent activation energy with the logarithm of the electric field. Published TDDB data for SiO_2_, high-k, low-k, and Cu/low-k dielectric systems exhibit this behavior. Independent temperature- and field-dependent measurements from the same source yield a common entropic field-energy scale of approximately 1.2 eV. A one-second reference field, $E_{1sec}$, provides a physical time anchor that allows field and temperature acceleration to be compared without assuming a common pre-exponential factor across temperatures. For an absolute time temperature series, independently extracted values of α vary by 11.9% across three temperatures, while a fixed α produces a normalized relation with R^2^ ≈ 0.97. The parameters $E_{a}$, α, and $E_{1sec}$ provide a compact thermodynamic representation of field- and temperature-dependent TDDB kinetics across the datasets examined here.

## 1. Introduction

Time-dependent dielectric breakdown (TDDB) is a major reliability limitation in semiconductor devices. Reliability prediction has traditionally relied on empirical acceleration models, including the multiplicative π-factor approaches used in reliability handbooks [1]. Temperature acceleration is commonly described by an Arrhenius relation with a fixed or representative activation energy [2]. However, measurements of TDDB over a range of electric fields show that the apparent activation energy is not constant but decreases systematically with increasing electrical stress [3].

This field dependence is commonly incorporated into lifetime models by allowing the activation energy or acceleration factor to depend explicitly on the electric field. Such models can reproduce the measured behavior, but the physical meaning of field-dependent activation energy remains unclear. In particular, an Arrhenius slope obtained from temperature-dependent lifetime measurements need not represent an intrinsic energy barrier when another thermodynamic variable changes with stress.

Device degradation can instead be described in terms of the Gibbs free energy of a metastable system [4]. Fabricated dielectric materials reside in metastable states and evolve toward states of lower Gibbs free energy [5]. The transition rate depends on both activation enthalpy and activation entropy. An applied electric field can therefore accelerate degradation without requiring the intrinsic enthalpic barrier to change. The field changes the number of accessible states and hence the entropy associated with the degradation process [6]. Within this description, the apparent activation energy obtained from an Arrhenius plot contains both the intrinsic energetic barrier and a field-dependent entropic contribution [7].

This separation leads to a simple experimental question. If the intrinsic activation energy is independent of the field and the field acceleration enters through entropy, the apparent activation energy should vary linearly with the logarithm of the electric field. The slope of this relation defines an entropic field parameter, α. A reference field can then be chosen without changing α, which allows measurements from different dielectric systems to be compared on a common basis. A second reference, the field $E_{1sec}$ corresponding to a one-second lifetime, provides a direct normalization of field and temperature acceleration [8].

In this work, published TDDB data from SiO_2_, high-k, low-k, and Cu/low-k dielectric systems are used to examine these relationships. The apparent activation energy is first compared with a logarithmic electric field. Independent temperature-dependent datasets are then used to compare the extracted field parameters, followed by an examination of $E_{1sec}$ normalization. The analysis is intended to determine whether field and temperature acceleration in these different TDDB datasets can be represented by a common thermodynamic form.

## 2. Thermodynamic Framework for Reliability Kinetics

TDDB is an irreversible transition from a metastable dielectric state toward a more stable degraded state [5]. The rate of this transition depends on the free-energy barrier separating these states. Both temperature and electric field affect the transition rate and must therefore be included in the thermodynamic description.

### 2.1. Gibbs Free Energy and Electrical Stress

The appropriate thermodynamic quantity for a transition at constant temperature and pressure is the Gibbs free energy. For an activated transition, the free-energy barrier can be written as follows:

(1) $$\Delta G^{‡}=\Delta H^{‡}-T\Delta S^{‡}$$

Here, $\Delta G^{‡}$ is the activation free energy, $\Delta H^{‡}$ is the activation enthalpy, T is the absolute temperature, and $\Delta S^{‡}$ is the activation entropy. The enthalpic term represents the energetic barrier associated with the transition. We identify this intrinsic barrier with the activation energy, $E_{a}$. The entropy term accounts for the number of states available to the system as it crosses this barrier.

The distinction between these two contributions becomes important under electrical stress. TDDB occurs in dielectric materials containing polar or ionic bonds [5]. An applied electric field polarizes the dielectric and changes the forces acting on these bonds [5]. Moreover, the field experienced locally by a bond need not equal the externally applied field. Local fields can be considerably larger, particularly in high-k materials with large Lorentz factors [5]. The applied field can therefore strongly influence the probability of a degradation transition even when the intrinsic energetic scale associated with the bond remains unchanged.

This distinction can be expressed statistically. Consider an intact dielectric configuration with $\Omega_{i}$ accessible microstates and a degraded configuration with $\Omega_{f}$ accessible microstates. Bond rupture and the accompanying structural rearrangement generally increase the number of configurations available to the system. The corresponding change in activation entropy is then given by the Boltzmann relation:

(2) $$\Delta S^{‡}=k\ln\left(\frac{\Omega_{f}}{\Omega_{i}}\right)$$

where k is the Boltzmann constant. Substituting the Gibbs free energy definition into the standard transition probability equation isolates the enthalpic and entropic contributions:

(3) $$\exp\left(-\frac{\Delta G^{‡}}{kT}\right)=\exp\left(-\frac{\Delta H^{‡}}{kT}\right)\exp\left(\frac{\Delta S^{‡}}{k}\right)$$

The first exponential term represents the energetic barrier to the transition, while the second represents the contribution from the increased number of accessible microstates. The relevant entropy is therefore not determined by the absolute number of configurations in either state but by the change in configurational multiplicity associated with the transition. An applied electric field may alter this difference by constraining the configurations accessible to the intact dielectric relative to those associated with the degradation transition. The field can therefore modify the activation entropy without supplying the energy required to break an individual chemical bond.

### 2.2. Field-Dependent Microstates and Field Acceleration

An external electric field, E, changes the number of configurations accessible to the degrading system. We link this change directly to statistical mechanics. The activation entropy relates to the initial ($\Omega_{i}$) and final ($\Omega_{f}$) configurational microstates through the Boltzmann equation. Electrical stress alters this configurational landscape. If the ratio of accessible microstates exhibits a power-law dependence on the applied electric field relative to a reference field $E_{0}$, we write this relation as follows:

(4) $$\Omega_{f}/\Omega_{i}\propto {\left(E/E_{0}\right)}^{\gamma }$$

where the exponent γ represents the dimensionless field acceleration parameter. Substituting this power-law dependence into the Boltzmann equation directly yields a logarithmic field dependence for the entropic contribution:

(5) $$\Delta S_{E}^{‡}=k\gamma \ln\left(E/E_{0}\right)$$

This derivation demonstrates that the logarithmic dependence arises naturally when the underlying microstate ratio responds to electrical stress via a power law. A large value of γ therefore need not imply a correspondingly large direct energetic interaction between the field and an individual bond. Instead, it indicates a strong collective configurational response of the dielectric to electrical stress. We represent this field dependence by allowing the activation entropy to vary logarithmically with the applied field relative to a reference field, $E_{0}$:

(6) $$\Delta S_{E}^{‡}=\left(\alpha /T\right)\ln\left(E/E_{0}\right)$$

where α has units of energy, $E_{0}$ is the reference electric field, and $T\Delta S_{E}^{‡}$ represents the field-dependent entropic contribution to the activation free energy. The corresponding field acceleration factor, γ, follows directly from the thermodynamic entropy parameter:

(7) $$\gamma =\frac{\alpha }{kT}\;\;\;.$$

This relation normalizes the entropic energy constant against the thermal energy of the system. The model does not assume that diverse dielectrics, such as SiO_2_ and HfO_2_, share a common bond polarization mechanism. These materials possess fundamentally different microscopic physics. These microscopic differences dictate the specific extracted values of the activation energy ($E_{a}$) and the entropic field parameter (α). However, the shared thermodynamic form remains consistent. The linear decrease in apparent activation energy with logarithmic electric field results naturally from the power-law response of the configurational microstates, rather than from a single specific bond-breaking interaction. Since α has units of energy, γ is dimensionless and varies inversely with temperature. The temperature dependence of the TDDB field exponent can therefore be written as follows:

(8) $$N\left(T\right)=N_{0}+\frac{\alpha }{kT}$$

where $N_{0}$ represents the temperature-independent component. We write the clean form of the lifetime relation as follows:

(9) $$\ln TTF={\ln C}_{0}+\frac{E_{a}}{kT}-\left(N_{0}+\frac{\alpha }{kT}\right)\ln\left(\frac{E}{E_{0}}\right)$$

where $C_{0}$ acts as a proportionality constant. We rearrange this equation to group the temperature-dependent terms:

(10) $$\ln TTF={\ln C}_{0}-N_{0}\ln\left(\frac{E}{E_{0}}\right)+\frac{E_{a}-\alpha \ln\left(E/E_{0}\right)}{kT}\;\;\;.$$

Equation (10) separates the fixed enthalpic contribution, $E_{a}$, from the field-dependent entropic contribution, $\alpha \;ln\left(E/E_{0}\right)$. The apparent activation energy obtained from the Arrhenius slope is therefore as follows:

(11) $$E_{a,app}\left(E\right)=E_{a}-\alpha \ln\left(\frac{E}{E_{0}}\right)\;\;\;.$$

Equation (11) predicts a linear decrease in apparent activation energy with $\ln\left(E/E_{0}\right)$. The choice of $E_{0}$ determines the intercept but does not change the slope. We demonstrate this independence by taking the derivative of the apparent activation energy with respect to the logarithmic field $\frac{dE_{a,app}}{d\ln E}=-\alpha$. This derivative depends only on the entropic parameter α and remains completely independent of $E_{0}$. We may therefore choose a new reference field, $E_{0,i}$, for each dataset such that the apparent activation energy reaches a common target value:

(12) $$E_{a,app}\left(E\right)=E_{a,target}-\alpha_{i}\ln\left(\frac{E}{E_{0,i}}\right)\;\;\;.$$

A separate reference field, $E_{0,i}$, can be calculated for each technology by choosing a common target activation energy:

(13) $$E_{0,i}=E_{0,ref}\exp\left[\frac{E_{a,i}-E_{a,target}}{\alpha_{i}}\right]\;\;\;\;.$$

Equation (13) gives the reference field required for each dataset to reach the selected activation energy value. Since this transformation is a horizontal translation on a logarithmic field scale, it leaves the slope $-\alpha_{i}$ unchanged. Different datasets can therefore be placed on a common reference-field scale without changing their extracted field sensitivity.

### 2.3. The 1-Second Reference Field

A standard one-second time to failure was chosen as the reference value for normalizing the measurements. This choice provides a specific temporal anchor for the thermodynamic framework. The reference electric field, $E_{1sec}$, corresponds to breakdown at exactly one second for a given temperature. The substitution of this temporal reference into the governing lifetime equation cancels the apparent activation energy terms and normalizes the time to failure by the entropic field acceleration factor:

(14) $$\ln\left(TTF\right)=-\left(\frac{\alpha }{kT}\right)\ln\left(\frac{E}{E_{1sec}}\right)\;.$$

Equation (14) provides a direct test of the proposed separation. For a thermally activated TDDB process described by a common value of α, lifetime data obtained at different temperatures should follow the same normalized relation when referenced to $E_{1sec}$. This representation avoids the need to assign a single global value of $N_{0}$ across the complete temperature range. Its applicability can therefore be examined directly using temperature-dependent TDDB data.

## 3. Literature-Based Extraction and Thermodynamic Parameterization

Published TDDB measurements provide an opportunity to examine the thermodynamic relations developed in Section 2 across different materials and experimental conditions. We compiled data for SiO_2_, high-k, low-k, and Cu/low-k dielectric systems obtained from several independent studies. These datasets span different electric fields, temperatures, technologies, and laboratories. Table 1 identifies the source and original figure for each dataset. The extracted parameters are compared using both field- and temperature-dependent measurements.

### 3.1. Field-Dependent Activation Energy and Parameter Extraction

Figure 1 shows the apparent activation energy as a function of the logarithmic electric field for the compiled datasets. Each dataset exhibits an approximately linear decrease in $E_{a,app}$ with increasing $\ln\left(E/E_{0}\right)$, as predicted by Equation (11). The intercept provides the reference activation energy, $E_{a}$, while the negative slope gives the entropic field parameter, α. The linear fits yield $R^{2}$ values ranging from 0.883 to 0.999. We calculated the 95% confidence intervals for the fitted parameters to quantify the regression uncertainty associated with the extracted data. Table 2 reports these explicit uncertainty bounds for $E_{a}$ and α.

### 3.2. Reference-Field Normalization

Equation (13) provides a reference-field transformation that places the different datasets on a common activation energy baseline. The target value was chosen as the median intrinsic activation energy of the compiled datasets. Figure 2 shows the resulting horizontal translation. Each dataset is shifted so that its extrapolated intercept reaches the selected target value at $ln\left(E/E_{0,i}\right)=0$, while its slope and corresponding value of $\alpha_{i}$ remain unchanged. The resulting $E_{0,i}$ is a mathematical reference coordinate used to compare the datasets and does not necessarily represent a physically measurable characteristic field of the dielectric. This distinguishes $E_{0,i}$ from $E_{1sec}$, which is defined by an experimentally measurable breakdown time.

Figure 3 shows the extracted $E_{a}$ and α values for the different technologies. The high-k materials generally exhibit larger values of α than conventional silicon dioxide, although the number of high-k datasets is limited. The low-k and Cu/low-k systems span a broader range. Table 2 summarizes the extracted parameters and the corresponding reference fields for all datasets.

### 3.3. Thermodynamic Cross-Validation

Figure 4 provides an independent Arrhenius analysis of the temperature dependence for five dielectric configurations reported in the literature. The extracted activation energies range from approximately 0.20 to 0.59 eV. Since these measurements were obtained from different technologies and experimental representations, they are not used for direct comparison with the $E_{a}$ values in Table 2. They provide an independent measure of the activation energy scale obtained from conventional temperature-dependent analysis.

A more direct comparison is possible using the same-source data shown in Figure 5. Apparent activation energies were extracted from temperature-dependent breakdown measurements at several voltages and plotted as a function of $ln\left(V/V_{0}\right)$. The resulting linear dependence gives α=1.162 eV with $R^{2}=0.966$. The field-dependent dataset from the same source (Dataset B03) gives α=1.258 eV. These two independent extractions therefore give a common entropic field-energy scale of approximately α=1.21 ± 0.05 eV, where the stated variation represents the spread between the two determinations rather than a statistical confidence interval. The regression uncertainty is considerably larger, particularly for the temperature-dependent extraction because of the limited number of voltage points. The corresponding 95% confidence intervals are α=1.162 ± 2.759 eV for the temperature-dependent extraction and α=1.258 ± 0.728 eV for the field-dependent extraction. The 0.096 eV difference between the two independently determined values lies within both confidence intervals.

Figure 6 examines the stress exponent $N\left(T\right)$ as a function of inverse temperature. Equation (8) predicts a positive slope when α is positive and $N_{0}$ is independent of temperature. The standard HfO_2_ dataset follows this behavior, whereas the FE-HfO_2_ dataset exhibits a negative apparent slope. The fits are based on only four and three temperature points, respectively, which limits the statistical confidence in the fitted slopes and in the inferred temperature dependence of $N\left(T\right)$. Figure 6 is therefore used to illustrate the observed dataset-specific behavior rather than to establish a general temperature law. The $E_{1sec}$ normalization introduced in Equation (14) provides a separate test that can be applied independently at each temperature.

.

### 3.4. Voltage Acceleration and Breakdown Field

Equation (7) relates the field acceleration factor, γ, to the entropic parameter through γ = α/kT. Figure 7 compares the extracted values of γ with reported breakdown fields. Figure 7A shows the Weibull representation of the available low-k breakdown-field data. Figure 7B compares the corresponding mean breakdown fields with γ at 298 K. Within the available data, larger values of γ align with lower breakdown fields. We treat this trend strictly as an exploratory correlation. The plotted data originate from different source experiments. These experiments differ significantly in dielectric composition, material thickness, device area, testing temperature, and breakdown criteria. These test condition differences systematically influence the observed trend.

### 3.5. The 1-Second Reference-Field Normalization

Equation (12) provides a direct normalization of the lifetime data using the reference field $E_{1sec}$. Figure 8 applies this normalization to the two available temperature-series datasets. For N_01_, which contains absolute $t_{BD}\;\left(63\%\right)$ values in seconds, the independently extracted values of α have a coefficient of variation of 11.9% across the three temperatures. A single fixed value of α then produces the normalized relation shown in Figure 8C, with R^2^ ≈ 0.97.

The N02 dataset provides an additional temperature series, but the reported breakdown times are normalized rather than given on an absolute time scale. It can therefore be used to examine the temperature dependence of the transformation but cannot define an absolute one-second reference field. The determination of $E_{1sec}$ from Equation (14) requires TTF reported on an absolute time scale.

## 4. Discussion and Conclusions

The literature data examined in Section 3 span different dielectric materials, electric fields, temperatures, and experimental methods. Despite these differences, the field and temperature dependencies can be described using a small set of thermodynamic parameters. The significance of this result is not that all TDDB data share the same parameters but that the differences among datasets can be represented within the same thermodynamic framework.

### 4.1. Discussion

The apparent activation energy of TDDB decreases with an increasing electric field for all of the datasets examined here. Within the present framework, this behavior follows from the separation of the field-independent activation barrier, $E_{a}$, and the field-dependent entropic contribution represented by α. The extracted values of both $E_{a}$ and α differ among dielectric technologies. These differences are expected because the parameters describe the particular material system and degradation process rather than universal constants.

The reference-field transformation provides a useful way to compare the different datasets without changing their field dependence. A change in $E_{0}$ translates the data along the logarithmic field axis while leaving the slope, and therefore α, unchanged. More importantly, the temperature- and field-dependent measurements from the same source in Figure 5 independently give a common entropic field-energy scale of approximately 1.2 eV. This agreement provides a direct connection between the temperature and field dependencies described by the thermodynamic framework.

The temperature dependence also shows why a fixed global N_0_ is not always sufficient. The standard HfO_2_ and FE-HfO_2_ datasets in Figure 6 do not follow the same $N\left(T\right)$ behavior over the measured temperature ranges. The one-second reference field provides an alternative normalization because $E_{1sec}$ can be determined independently at each temperature without assuming a common $N_{0}$. For the absolute time *N*_01_ dataset, the independently extracted values of α remain within an 11.9% coefficient of variation over the three temperatures, and a fixed value of α gives R^2^ ≈ 0.97 after normalization.

The large magnitude of γ observed for TDDB is also significant. Since γ = α/kT, values approaching 40 do not imply that the applied field transfers a correspondingly large energy to an individual chemical bond. Rather, α represents the energy scale associated with the field-dependent change in configurational entropy. Within this interpretation, increasing electrical stress progressively constrains the microscopic configurations accessible to the metastable dielectric, thereby reducing the entropic contribution to the free-energy barrier separating the intact and degradation-compatible states. A large value of γ therefore indicates a strong collective configurational sensitivity to electrical stress rather than direct field-induced bond dissociation.

SiC MOSFET gate oxides provide an interesting application of this framework. Under dynamic switching conditions, the electric field across the oxide depends on the voltage-dependent device capacitances and switching waveform [18]. Field- and temperature-dependent SiC TDDB measurements could therefore be analyzed using the same thermodynamic formulation to extract $E_{a}$ and α. Such measurements would provide a useful test of whether the entropic field dependence observed here extends to SiC gate oxide degradation under dynamic operating conditions.

Finally, the comparison between γ and breakdown field in Figure 7 suggests that materials with stronger field acceleration may also have lower breakdown fields. The present cross-source dataset is too limited to establish a causal relationship, but the observed trend provides a useful question for future experiments in which γ and EBD are measured on the same dielectric structures.

### 4.2. Limitations

The present analysis is based on published data rather than measurements obtained in a single controlled experiment. This provides access to several dielectric technologies and experimental conditions, but it also introduces uncertainty associated with digitization and differences among the original measurements. The 95% confidence intervals in Table 2 describe the regression uncertainty of parameters extracted from the digitized data; they do not separately quantify the digitization error. Some datasets also required mathematical conversion before their parameters could be compared.

The source datasets originate from different experimental setups. The tested devices differ in dielectric thickness, device area, and structural geometry. The experimental protocols also employ different stress waveforms, temperature ranges, Weibull percentiles, and failure definitions. These differences can introduce systematic offsets in the extracted values of $E_{a}$ and α.

The number of available datasets limits conclusions about individual material classes. The high-k, SiO_2_, low-k, and Cu/low-k groups contain too few independent datasets to establish general technology trends. Furthermore, some individual datasets contain a limited number of data points. These limited datasets remain useful for parameter extraction, but they do not establish definitive material properties. Therefore, Table 2 intentionally presents different parameters for each dataset. These values characterize the individual datasets and do not represent universal constants for SiO_2_, HfO_2_, or any other dielectric class.

The FE-HfO_2_ dataset exhibits the opposite temperature dependence from conventional HfO_2_. Ferroelectric HfO_2_ differs in structural phase and includes coupling between the applied field and internal polarization, either of which may alter the extracted field dependence. The apparent field exponent may therefore contain contributions not represented by the configurational entropy of Equation (6). The available literature data do not distinguish among these possible microscopic contributions, and a single temperature-independent $N_{0}$ does not describe both datasets over the measured temperature ranges.

$E_{1sec}$ normalization also requires breakdown times reported on an absolute time scale. Normalized $T_{BD}$ data examine the temperature dependence of the transformation, but they cannot provide an absolute one-second reference field. Controlled experiments designed specifically to extract $E_{a}$, α, γ, and $E_{1sec}$ on identical test structures remain an important next step. Such experiments would provide a direct test of the relationships examined here.

### 4.3. Conclusions

TDDB field and temperature acceleration can be described thermodynamically by separating the field-independent activation barrier, $E_{a}$, from the field-dependent entropic contribution characterized by α. Published TDDB datasets from several dielectric technologies exhibit the predicted decrease in apparent activation energy with the logarithm of the electric field. The extracted values of $E_{a}$ and α differ among dielectric systems, while changes in the reference field translate the field scale without changing α.

Independent temperature- and field-dependent measurements from the same source give a common entropic field-energy scale of approximately 1.2 eV. The one-second reference field, $E_{1sec}$, provides an absolute time anchor without requiring a common pre-exponential factor across temperatures. For the absolute time temperature series, independently extracted values of α have an 11.9% coefficient of variation over three temperatures, while a fixed value of α gives $R^{2}\;\approx \;0.97$ after normalization.

The parameters $E_{a}$, α, and $E_{1sec}$ therefore provide a compact thermodynamic representation of the field and temperature dependence observed in the TDDB datasets examined here. Their numerical values remain specific to the dielectric system and experimental conditions.

## Figures and Tables

**Figure 1:**
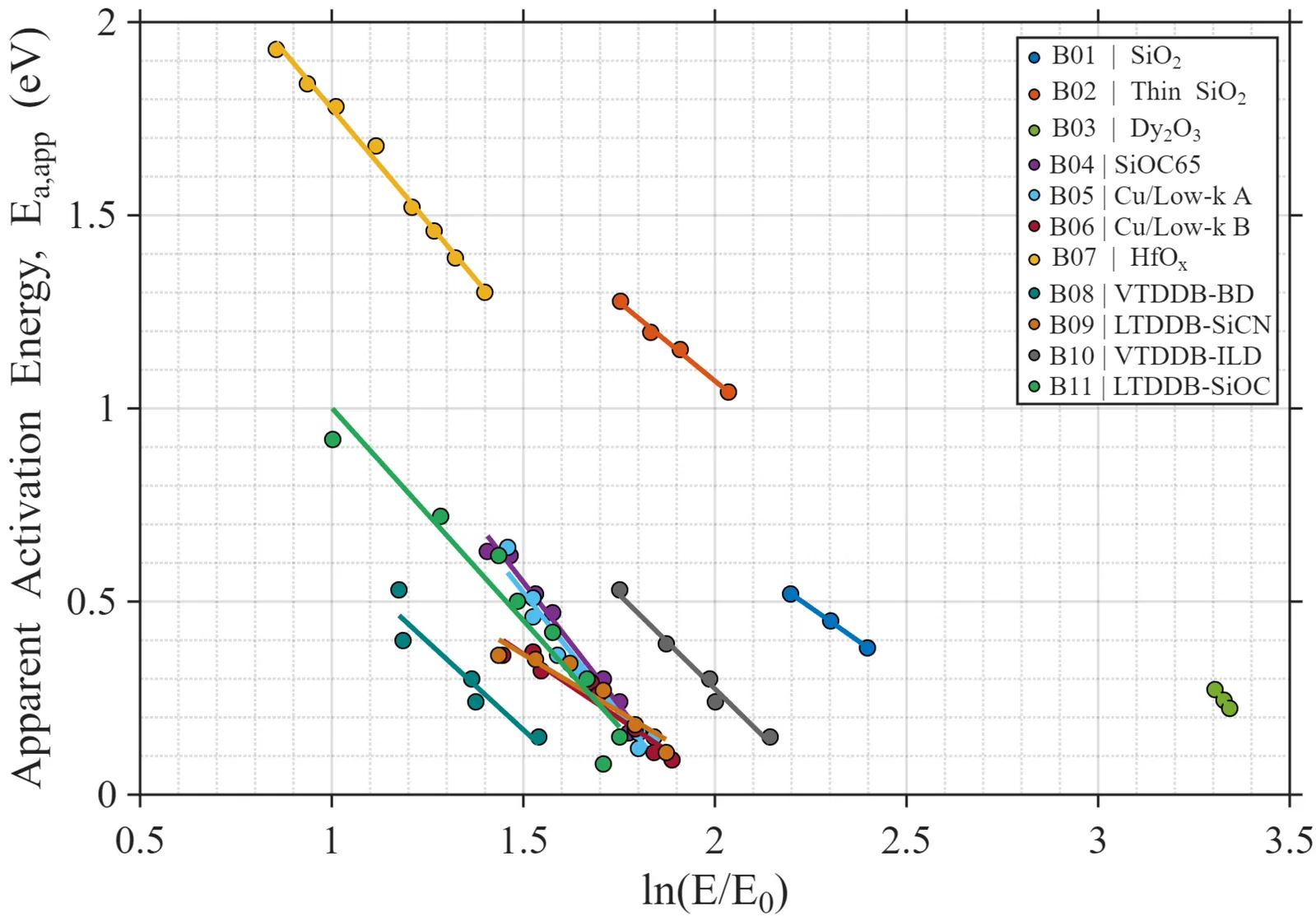
Field-dependent apparent activation energy.

**Figure 2:**
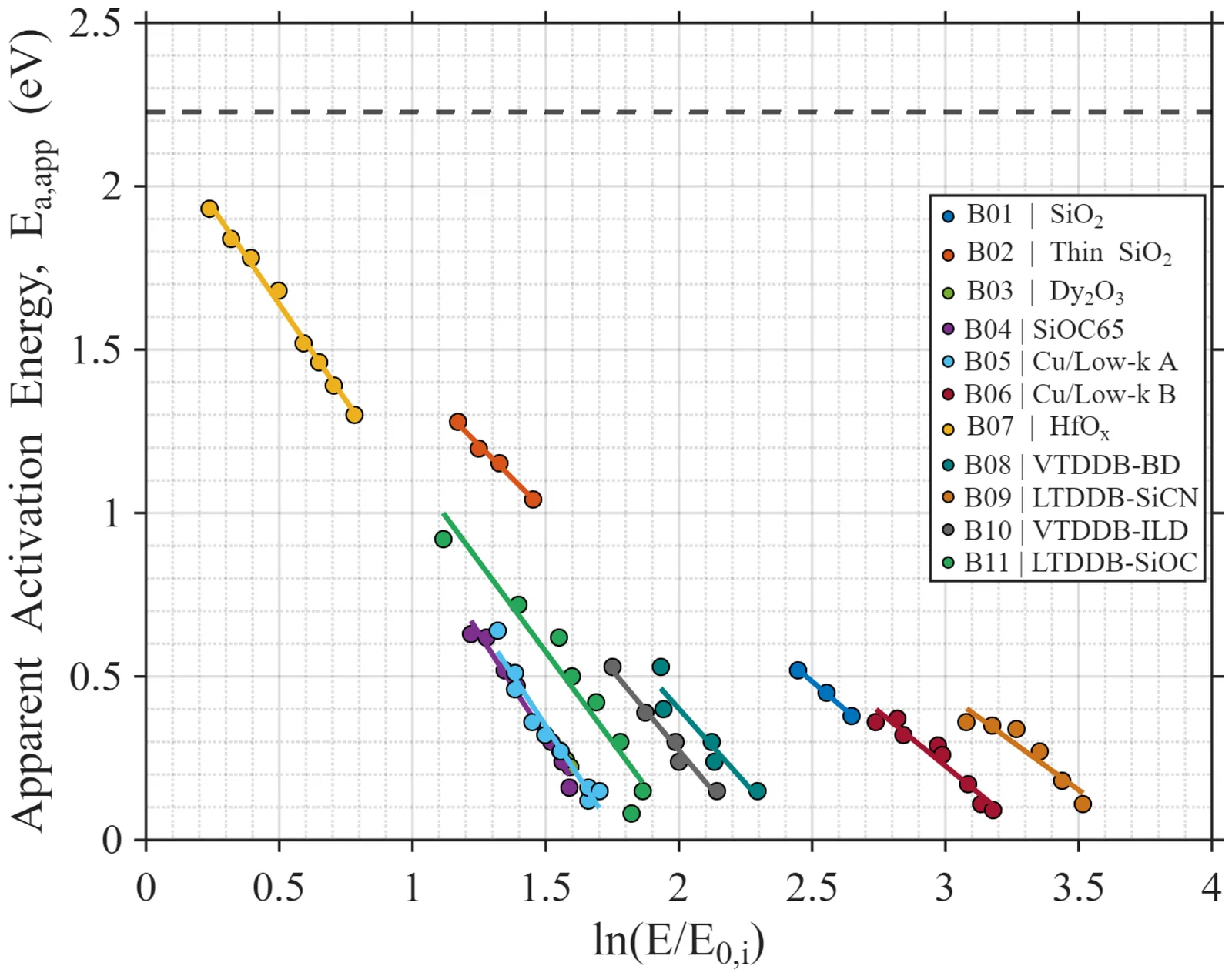
Reference-field horizontal collapse. The horizontal dashed line represents the chosen target activation energy. The mathematical transformation shifts each dataset horizontally so that its extrapolated intercept exactly meets this common thermodynamic baseline at $\ln\left(E/E_{0,i}\right)=0$.

**Figure 3:**
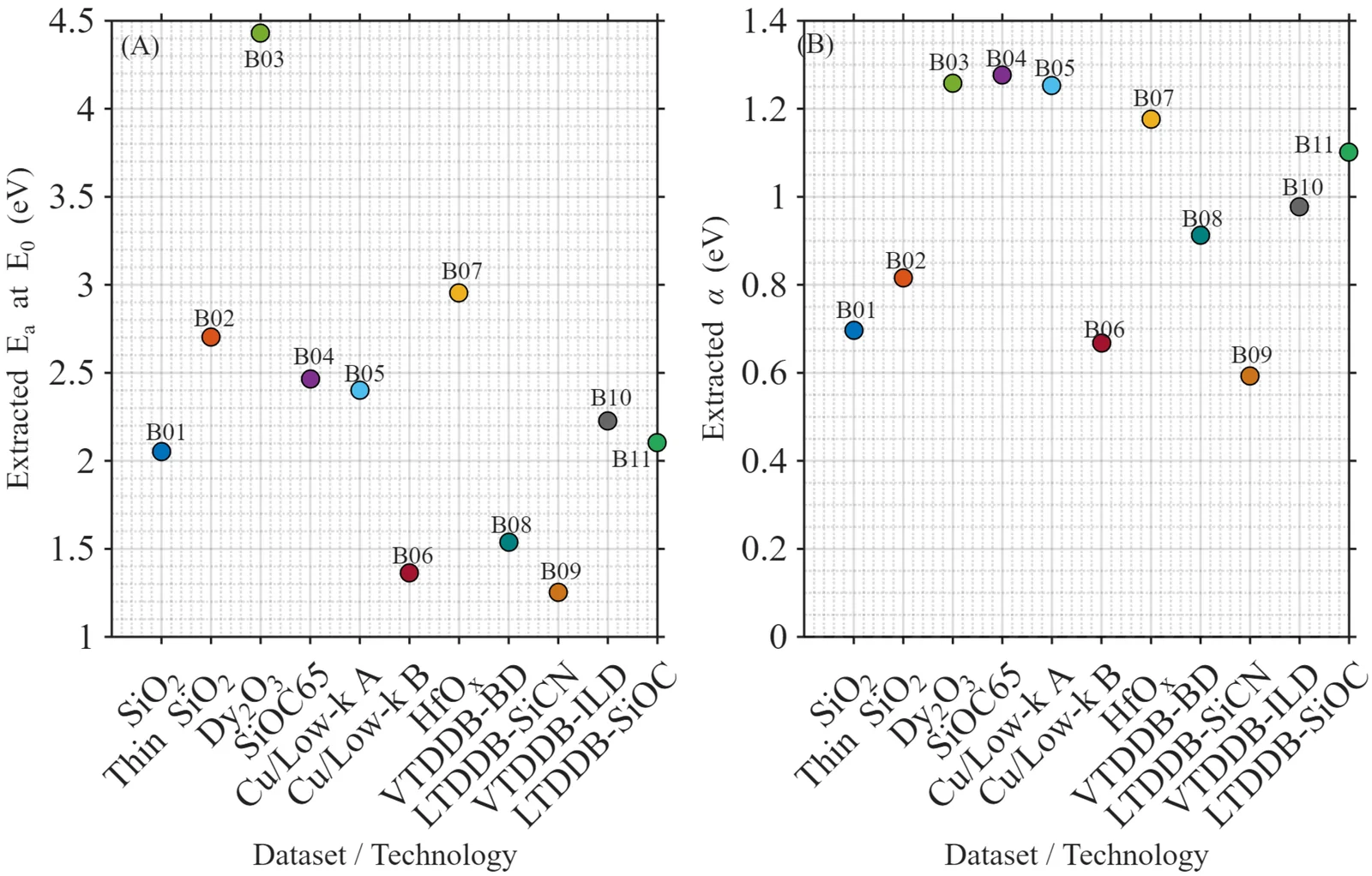
Technology dependence of $E_{a}$ and α: (**A**) extracted fixed $E_{a}$ and (**B**) field acceleration parameter α.

**Figure 4:**
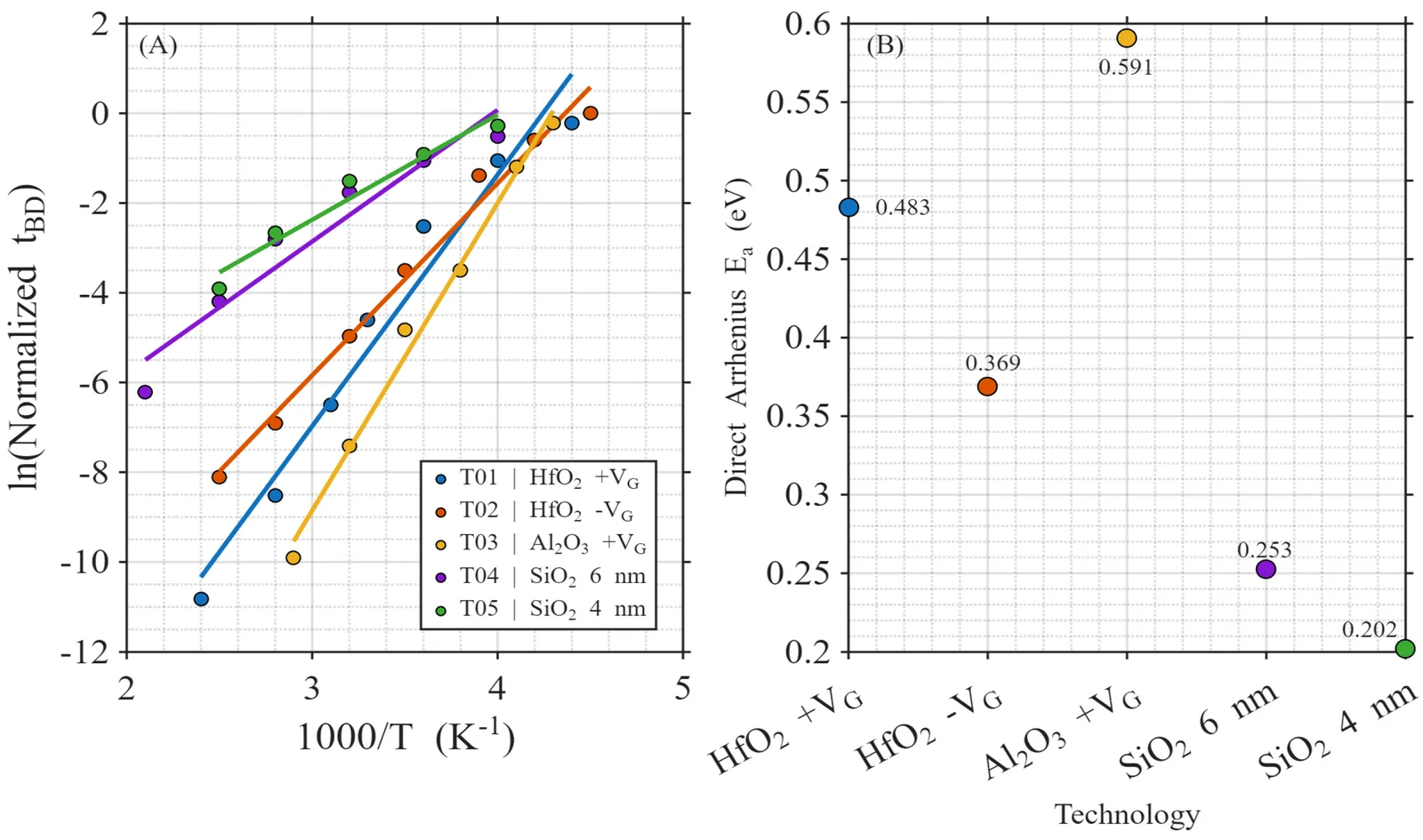
Direct Arrhenius activation energy: (**A**) direct Arrhenius extraction and (**B**) technology-level $E_{a}$.

**Figure 5:**
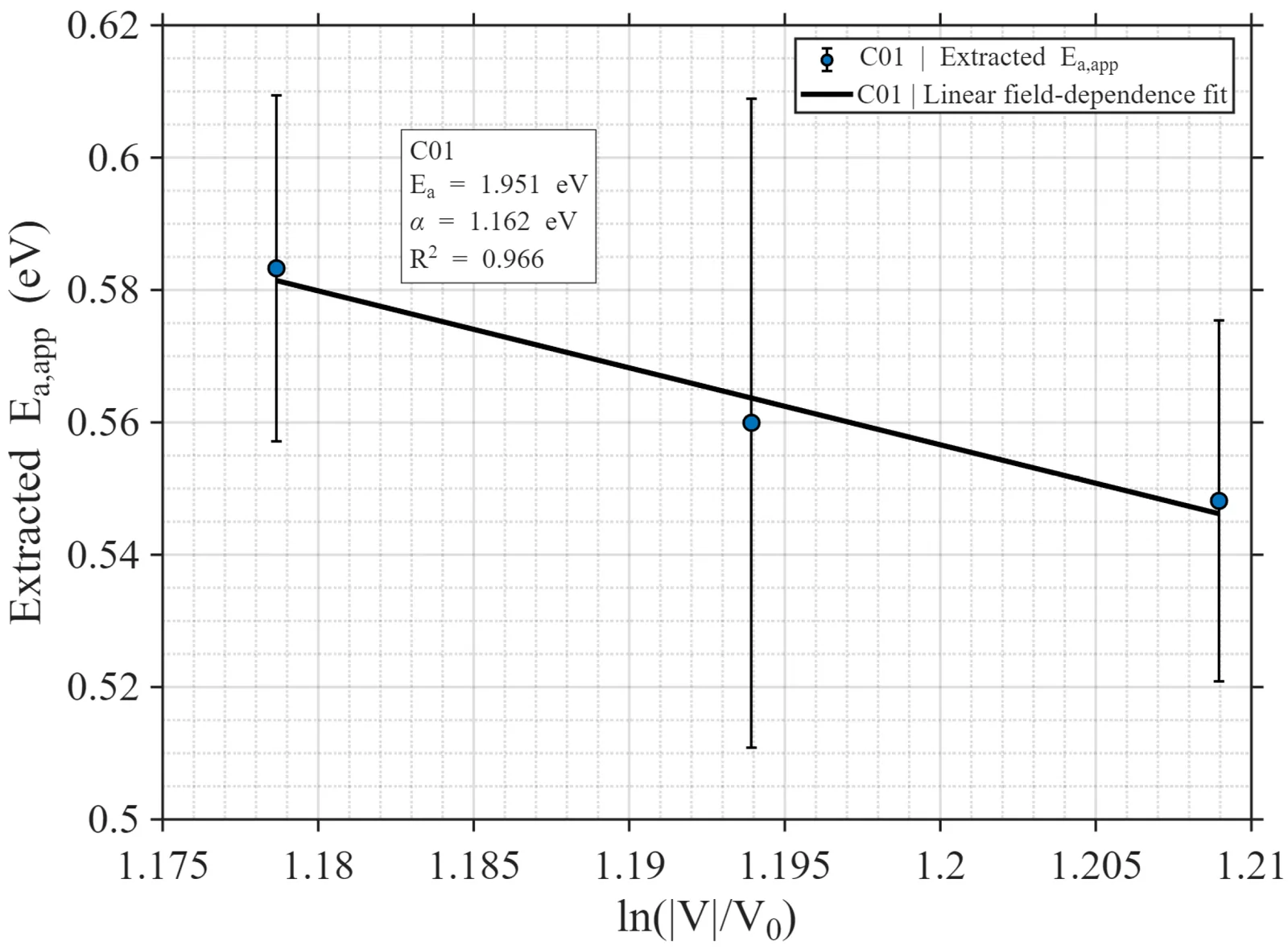
Same-source cross-validation.

**Figure 6:**
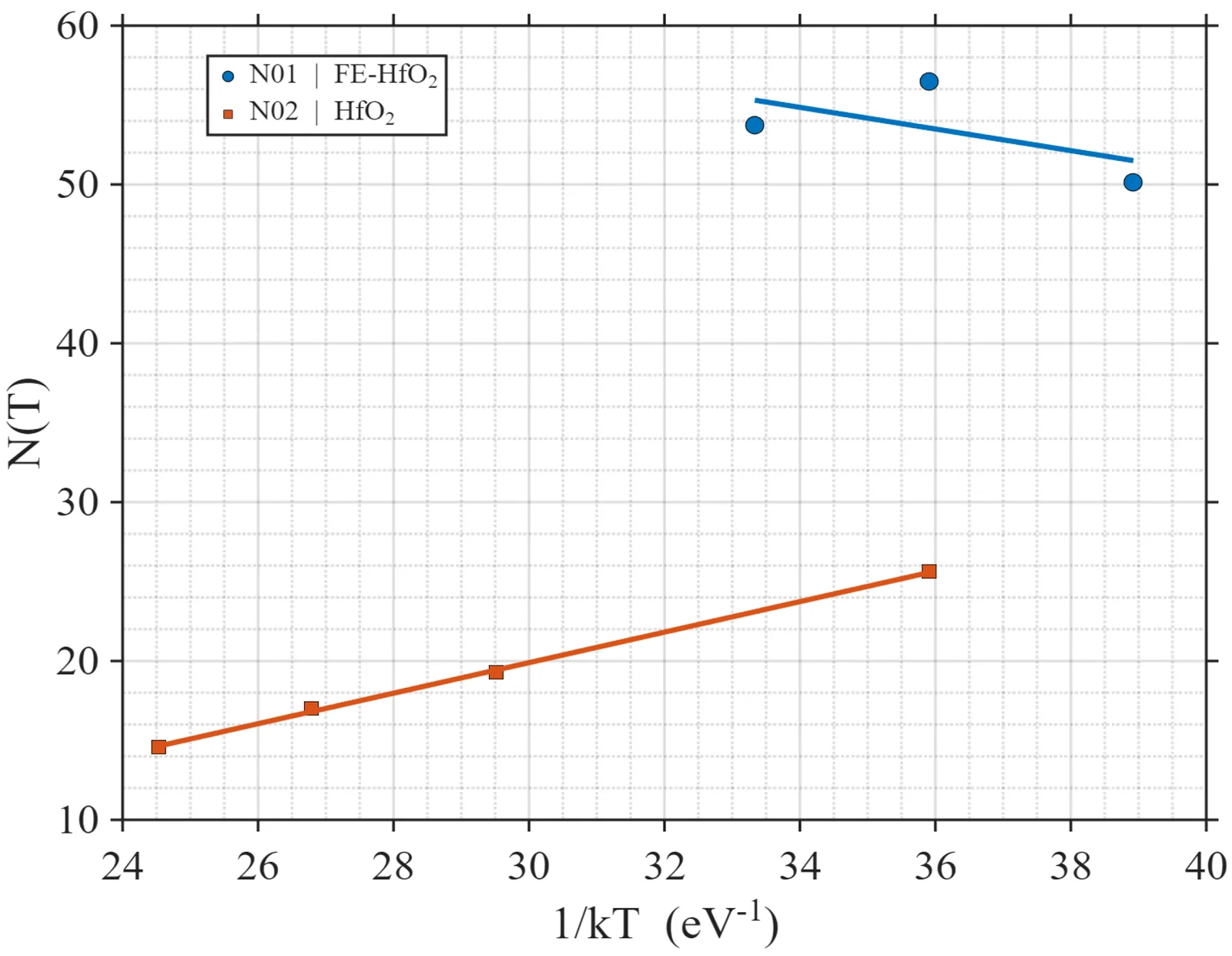
Temperature dependence of stress exponent N(T).

**Figure 7:**
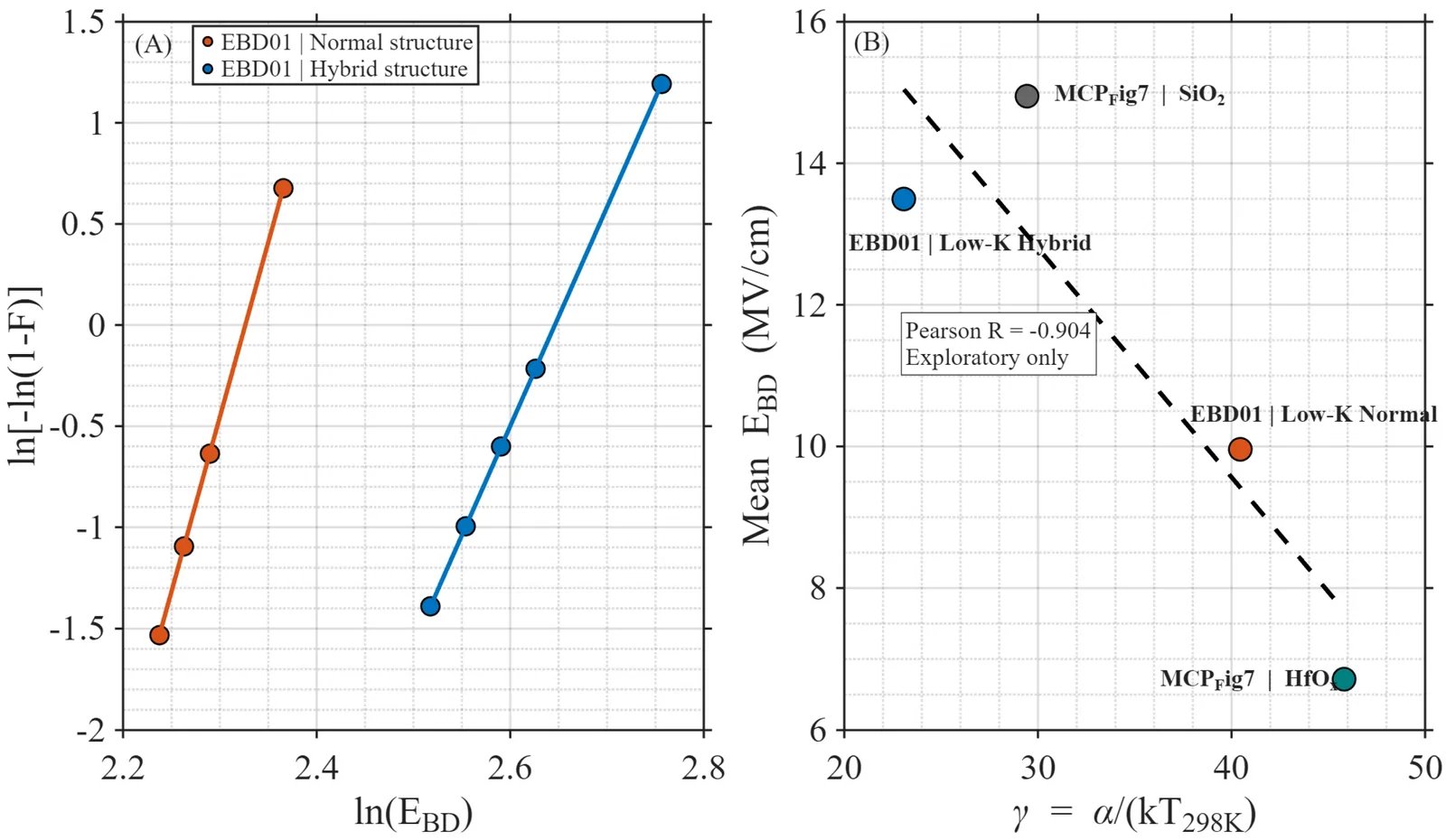
Breakdown field versus field acceleration parameter: (**A**) low-K breakdown-field Weibull reference and (**B**) exploratory $E_{BD}$ versus γ.

**Figure 8:**
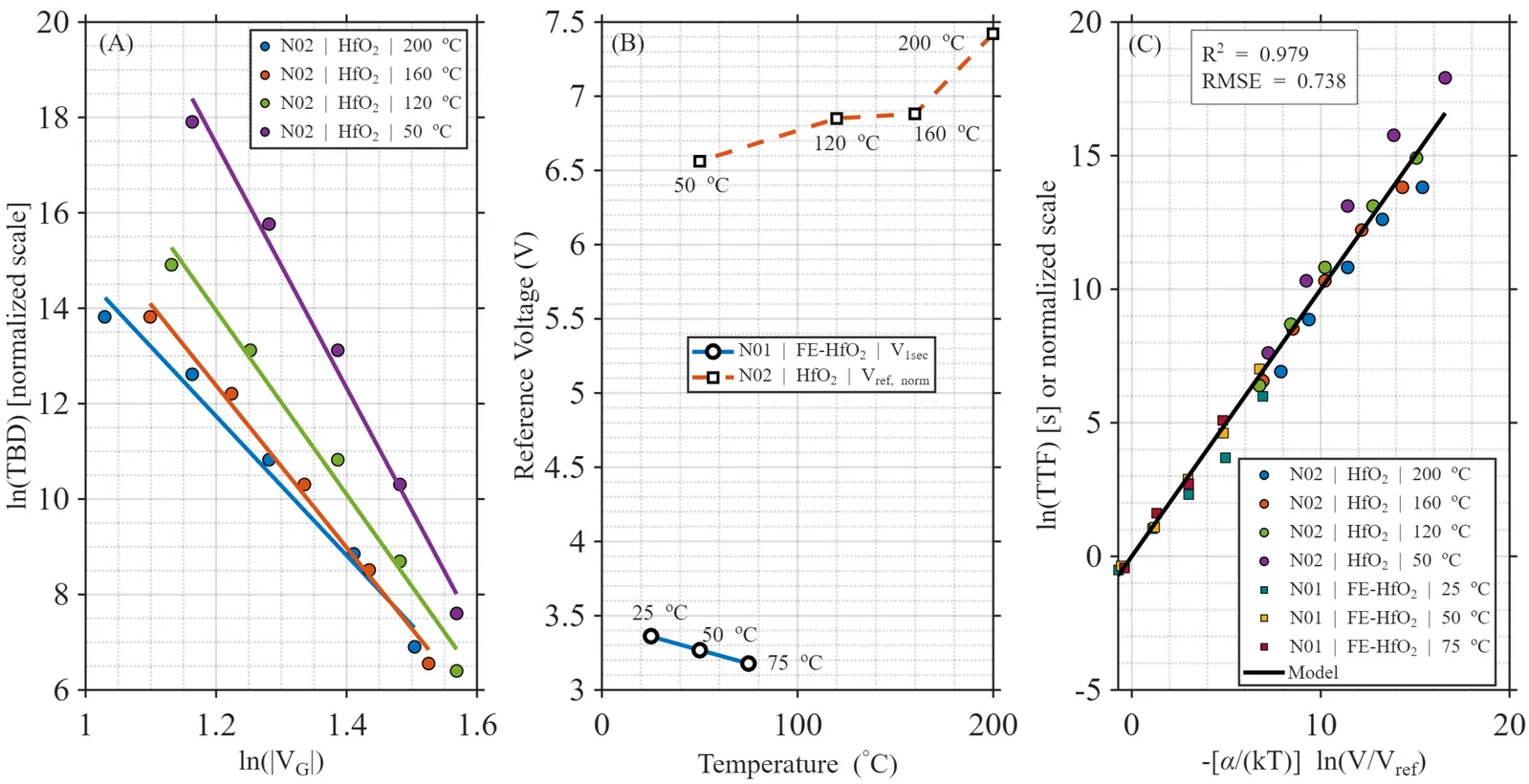
Normalized thermodynamic collapse—$E_{1sec}$: (**A**) temperature-dependent raw data, (**B**) reference stress versus temperature and (**C**) thermodynamic collapse.

**Table 1:** Literature datasets and provenance used for parameter extraction.

Dataset ID	Dataset Name	Source Paper	Source Figure	Input Data
B01	${\mathrm{SiO}}_{2}$	[9]	Figure 9	E
B02	Thin ${\mathrm{SiO}}_{2}$	[10]	Figure 4	E
B03	${\mathrm{Dy}}_{2}O_{3}$	[11]	Figure 12	$\sqrt{E}$
B04	SiOC65	[12]	Figure 10	$\sqrt{E}$
B05	Cu/Low-k A	[12]	Figure 7	E
B06	Cu/Low-k B	[12]	Figure 7	E
B07	${\mathrm{HfO}}_{x}$	[13]	Figure 4	E
B08	VTDDB-BD	[14]	Figure 7A	$\sqrt{E}$
B09	LTDDB-SiCN	[14]	Figure 7A	$\sqrt{E}$
B10	VTDDB-ILD	[14]	Figure 7B	$\sqrt{E}$
B11	LTDDB-SiOC	[14]	Figure 7B	$\sqrt{E}$
T01	${\mathrm{HfO}}_{2}\;+\;V_{G}$	[15]	Figure 1	1000/T, *Norm.* $T_{BD}$
T02	${\mathrm{HfO}}_{2}\;-\;V_{G}$	[15]	Figure 1	1000/T, *Norm.* $T_{BD}$
T03	${\mathrm{Al}}_{2}O_{3}\;+\;V_{G}$	[15]	Figure 1	1000/T, *Norm.* $T_{BD}$
T04	${\mathrm{SiO}}_{2}$ 6 nm	[15]	Figure 1	1000/T, *Norm.* $T_{BD}$
T05	${\mathrm{SiO}}_{2}$ 4 nm	[15]	Figure 1	1000/T, *Norm.* $T_{BD}$
C01	${\mathrm{Dy}}_{2}O_{3}/{\mathrm{HfO}}_{2}$ same-source	[11]	Figure 11	1000/T, $t_{BD}$
N01	FE-${\mathrm{HfO}}_{2}$ temp series	[16]	Figure 10	V, $t_{BD}\;\left(63\%\right)$
N02	${\mathrm{HfO}}_{2}$ temp series	[15]	Figure 3	$\left|V_{G}\right|$, *Norm.* $T_{BD}$
S01	${\mathrm{CuAl}}_{2}/{\mathrm{SiO}}_{2}$	[17]	Figure 6	1/kT, TTF
S02	${\mathrm{CuAl}}_{2}/{\mathrm{SiO}}_{2}$ field sweep	[17]	Figure 5	$\sqrt{E}$, TTF
EBD01	Cu/Low-k Normal/Hybrid	[14]	Text	$E_{BD}$ Weibull
MCP_Figure 7	$E_{BD}vs.\epsilon_{r}$	[5]	Figure 7	$\epsilon_{r}$, $E_{BD}$
MCP_Figure 6	$E_{BD}$ Dumin. $t_{ox}$	[5]	Figure 6	$t_{ox}$, $E_{BD}$

**Table 2:** Extracted activation and field acceleration parameters from literature datasets.

Dataset ID	Technology	$E_{a}\;\pm \;95\%\;\mathrm{CI}$ eV	α ± 95% CI eV	R^2^	RMSE eV	E_0_ Collapse MVcm
B01	${\mathrm{SiO}}_{2}$	2.053 ± 0.589	0.697 ± 0.256	0.999	0.002	0.778
B02	Thin ${\mathrm{SiO}}_{2}$	2.703 ± 0.403	0.816 ± 0.214	0.992	0.007	1.791
B03	${\mathrm{Dy}}_{2}O_{3}$	4.430 ± 2.420	1.258 ± 0.728	0.997	0.001	5.760
B04	SiOC65	2.466 ± 0.346	1.277 ± 0.215	0.979	0.025	1.205
B05	Cu/Low-k A	2.403 ± 0.427	1.253 ± 0.258	0.949	0.038	1.150
B06	Cu/Low-k B	1.365 ± 0.306	0.667 ± 0.182	0.930	0.027	0.274
B07	${\mathrm{HfO}}_{x}$	2.953 ± 0.113	1.177 ± 0.098	0.993	0.018	1.852
B08	VTDDB-BD	1.538 ± 0.783	0.913 ± 0.586	0.891	0.043	0.469
B09	LTDDB-SiCN	1.253 ± 0.500	0.593 ± 0.300	0.882	0.032	0.193
B10	VTDDB-ILD	2.227 ± 0.507	0.977 ± 0.259	0.979	0.019	1
B11	LTDDB-SiOC	2.103 ± 0.468	1.101 ± 0.310	0.926	0.073	0.892

## Data Availability

The original data presented in the study are openly available in IEEE Xplore at references [9,10,11,12,13,14,15,16,17] and in reference [5].
